# On the Possible Detection of Lightning Storms by Elephants 

**DOI:** 10.3390/ani3020349

**Published:** 2013-04-18

**Authors:** Michael C. Kelley, Michael Garstang

**Affiliations:** 1 School of Electrical and Computer Engineering, Cornell University, Ithaca, NY 14853, USA; E-Mail: mikek@ece.cornell.edu; 2 Department of Environmental Sciences, University of Virginia, Charlottesville, VA 22904, USA

**Keywords:** elephant communication, elephant detection of abiotic sounds, sounds from thunderstorms, elephant movement, elephant migration

## Abstract

**Simple Summary:**

We use data similar to that taken by the International Monitoring System for the detection of nuclear explosions, to determine whether elephants might be capable of detecting and locating the source of sounds generated by thunderstorms. Knowledge that elephants might be capable of responding to such storms, particularly at the end of the dry season when migrations are initiated, is of considerable interest to management and conservation.

**Abstract:**

Theoretical calculations suggest that sounds produced by thunderstorms and detected by a system similar to the International Monitoring System (IMS) for the detection of nuclear explosions at distances ≥100 km, are at sound pressure levels equal to or greater than 6 × 10^−3^ Pa. Such sound pressure levels are well within the range of elephant hearing. Frequencies carrying these sounds might allow for interaural time delays such that adult elephants could not only hear but could also locate the source of these sounds. Determining whether it is possible for elephants to hear and locate thunderstorms contributes to the question of whether elephant movements are triggered or influenced by these abiotic sounds.

## 1. Introduction

Sound in a highly social animal, such as an elephant, plays a crucial role. Sound influences survival of the species through communication, reproduction, resource utilization, and predation avoidance.

**Figure 1:**
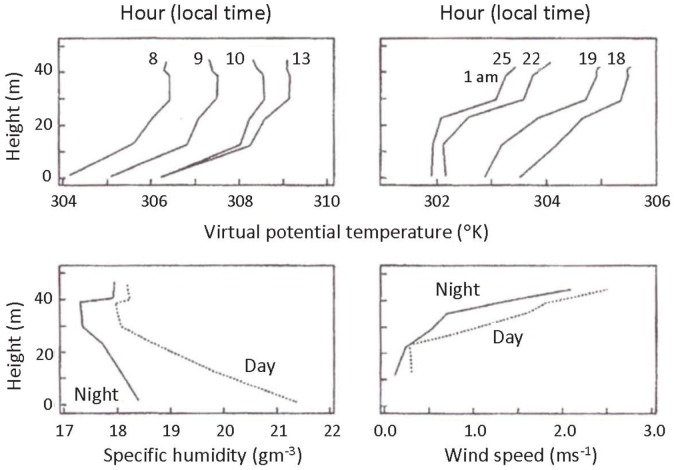
Mean values of rain forest meteorological conditions. Mean profiles of virtual potential temperature, *θ_v_* (°K) (top panels), specific humidity, *q* (gm^−3^) (night is solid, day is dotted) (lower left-hand side), and wind speed, *U* (ms^−1^) (night is solid, day is dotted) (lower right-hand side) within a 45 m high rainforest. The *θ_v_* profiles are identified by the hour of day over which they were averaged (12 = 1200; 25 = 0100, local time) [1].

The role of sound in the life of elephants can be traced back to their evolution in forests. Sight in a forest is of little value, and the distance over which smell can be used is limited by low levels of wind speed, as indicated in Figure 1, and by dispersion [1].

Low-frequency, long-wavelength sound, on the other hand, suffers little attenuation due to the presence of trees. The vertical thermal structure of temperatures in a forest finds the lowest temperatures at the floor and the highest temperatures at the top of the canopy (Figure 1). This is especially true in the daytime, resulting in a temperature inversion that creates a duct along which sound is channeled. Low wind speeds and low wind shear (changes in wind with height) result in either no or very low levels of turbulence (Figure 1). In the absence of wind and turbulence, sound is transmitted over significant distances, even in a forest setting. 

As elephantidae evolved in the forests some 35 million years ago, their highly social system was favored by these conditions. Males are ejected from the herd at puberty. While a female comes into estrous only 4 or 5 days every four years, mate selectivity could be exercised by reaching a number of males through the distant signaling of low-frequency estrous calls [1,2]. 

This ability to communicate enabled herds to avoid predators and enhanced the use of resources, enabling these large herbivores to consume some 150 kg of vegetation and find the huge amounts of water needed daily. Development of communication and communication strategies likely increased their brain capability, as is thought to be the case for other species, including humans [3]. 

**Figure 2:**
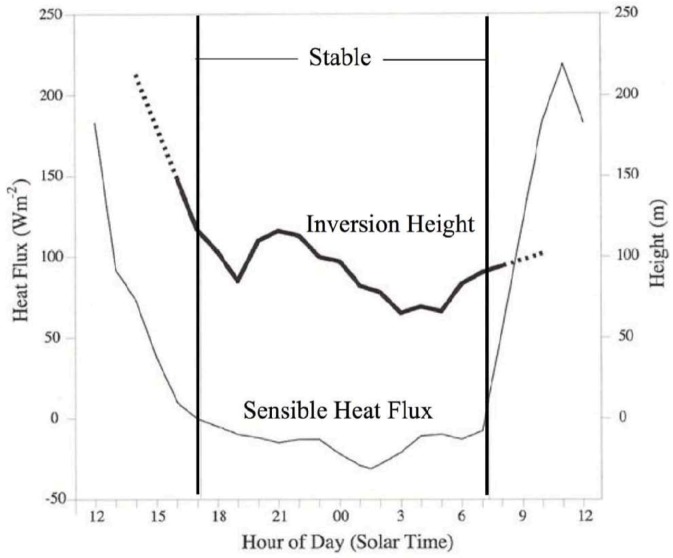
Savannah measurements of heat flux and inversion height. Sensible heat flux and inversion height over 24 hours over an open subtropical savanna. Mean values are shown from observations taken by a flux tower, vertical pointing, continuously operating SODAR and a profiling tethered balloon. The heavy line indicates a clearly defined inversion top, with dashes indicating disappearance of an inversion. The dashed line shows sensible heat flux (left ordinate) with the vertical lines delineating the stable region of negative heat flux at the surface [4].

When the subtropical savannas appeared and elephants moved from the shrinking forests to these open plains, atmospheric conditions during part of the 24-hour cycle resembled those of the forests. As shown in Figure 2, the presence of strong nocturnal temperature inversions allowed the elephants to utilize their long-distance, low-frequency capability to the fullest in this very different environment [2]. Note that in the presence of a stratified near-surface atmosphere (nocturnal inversion), the velocity field at higher altitudes is decoupled from the surface wind, resulting in very low or calm surface wind speeds. 

The ability of elephants to use sound goes well beyond direct communication within the species. Elephants can detect and use abiotic sources of sound in ways that have not been explored or understood. As a first step, this paper will determine whether a particular abiotic sound exists and is at sound pressure levels and frequencies that can potentially be detected and located by elephants.

## 2. Methods

**Figure 3:**
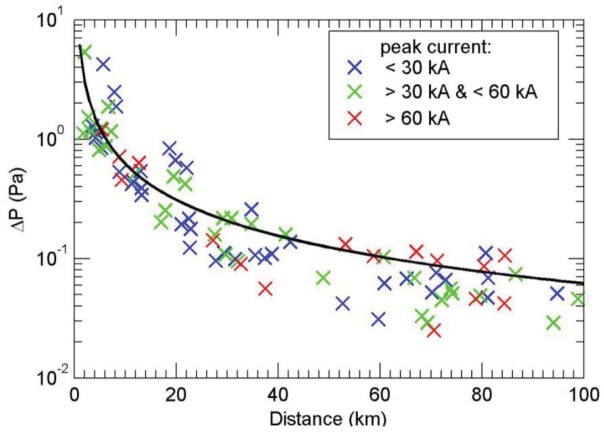
Lightning-generated infrasound amplitude *versus* ground distance. Amplitude of infrasound from lightning *versus* lightning distance (colored crosses) in a semi-log plot. The black line is the result of linear regression [5].

Sensible heat flux and inversion heights were taken from observations in typical elephant habitat by a flux tower, vertical pointing, continuously operating SODAR and a profiling tethered balloon (Figure 2). The infrasound data were obtained using instrumentation similar to that used by the International Monitoring System (IMS) infrasound network (Figure 3) and published by Farges and Blanc [5].

Evidence exists suggesting that elephants can detect distant thunderstorms heralding the end of the dry season [6]. Here we use the data from Farges and Blanc [5] and Farges *et al.* [7] to show that elephants can potentially hear sound generated by such storms from distances over 150 km. These distant thunderstorms represent many days (10–20) of travel for elephant herds. Sudden changes in herd movement may be in response to such storms and may signal the beginning of seasonal migrations [8]. 

Tracking of Global Positioning System (GPS) collared elephants over a period of seven years in Namibia has shown that there is a statistically significant change in the movements of herds during the dry season compared to their movements during the wet season [8]. This change in herd movement occurs suddenly at a time apparently related to the transition date from dry to wet conditions. The elephants responding in this fashion are far removed (≥100 km) from the first thunderstorms which were located by satellite. While Davis *et al.* [8] did not find a consistent time interval of response between the first occurrence of rain and the day on which movement pattern changed, the change in movement occurs without rain falling at the location of the elephants. The response to the distant thunderstorms would thus appear to be to some remote *versus* direct stimulus. Our intent, in this paper, is thus to explore the possibility that a signal generated by the distant thunderstorms can be detected at some distant location by the elephants. Detection of this signal could then trigger a change in the pattern of movement from one characteristic of the dry season to one which is characteristic of the wet season.

## 3. Detection of Sounds from Thunderstorms

Elephants use infrasound down to frequencies of 10 Hz and possibly lower [4,9]. Langbauer *et al.* [10] successfully transmitted signals to wild savannah elephants at a dB level of −6 dB at a distance of 2 km. Because the playback signals were transmitted at one-half power, Langbauer *et al.* [10] estimated that the range over which these calls could be heard could be doubled to 4 km. Alternatively, at 2 km animal, could easily detect −3 dB. The dB unit, *I*, is related to the threshold of human hearing, *P*_0_ = 2 × 10^−5^ Pascals (Pa), at 1,000 Hz by

*I*(dB) = 20log_10_(*P*/*P*_0_)

Thus, the received pressure pulse at 2 km is at least 14.2 Pa. The spherical (1/*r*^2^) intensity decay implies an initial squared pressure pulse at 1 m distance of 90 dB. In fact, Poole *et al.* [11], at this distance, measured sound pressure levels of up to 117 dB at frequencies between 14 and 35 Hz generated by an unstressed female elephant. 

The Langbauer *et al.* [10] measurements were carried out during the middle of the day. Under near optimum atmospheric acoustic conditions, which occur pervasively over open elephant savanna habitat, nocturnal inversions of temperature create a duct with a depth of 100 m (Figure 2) that channels the infrasound signal in a layer with calm or very low wind speeds [2,4]. This means that the squared pressure pulse (*P*^2^) falls off as 1/*r* rather that 1/*r*^2^. Thus, *P*^2^ at 10 km is on the order of 200/2*πrh* (Pa), where *h* is the depth of the inversion (100 m, see Figure 2). The acoustic pressure at 10 km is thus at least 6 × 10^−3^ Pa. 

With the threshold for human hearing at 1 kHz at 2 × 10^−5^ Pa, humans could hear these sounds [12,13]. It follows that elephants with far more sensitive hearing than humans, will be capable of detecting sounds much lower than 2 × 10^−5^ Pa. We note that the knowledge of the threshold of hearing of elephants is limited based on deduction from a single female, Asian elephant [14]. It is for this reason that we make the above comparisons to the threshold of human hearing and then relate human hearing to that of elephants.

To detect thunderstorm-generated infrasound, we use the results found by Farges and Blanc [5] and Farges *et. al.* [7]. At 100 km from a thunderstorm, they report the sound pressure to be 6 (±4) × 10^−2^ Pa with a spectrum that peaks just below 1 Hz and a power law spectrum varying as f^−0.3^ for 1–5 Hz. The conservative estimates detailed above thus suggests that elephants can hear infrasound generated by thunderstorms from very long distances from the source. Even with an *r*^2^ dependence with distance, the signal will be above 10^−5^ Pa at distances of hundreds of km. The recent unpublished 10-yr record [7] supports the earlier work with more robust statistics.

The question then arises as to whether elephants can determine the direction to the source of the sound. A rule of thumb for direction finding is that the sensors used must be separated by the order of a signal wavelength. For example, human ears are capable of direction finding for wavelengths less than about 0.1 m, corresponding to a few kHz. For elephants with sensor separations of a few meters, the frequency only needs to be greater than 100 Hz. However, such high-frequency acoustic signals will not propagate as far as infrasound, and direction finding will be limited to shorter distances than detection itself. Speculation here needs to be replaced by specific measurements to determine the direction finding capabilities of elephants. 

## 4. Conclusions

Elephants are capable of detecting thunderstorms at distances well over 100 km and, in principle, can find the storm azimuth as well. With experience, the sound intensity could, we believe, be used to gauge the distance to the thunderstorms as well. This hypothesis could be tested first under controlled conditions in zoos and sanctuaries, followed by the analysis of GPS-collared elephants and satellite-observed storm distributions. Infrasound data are readily available from the international nuclear monitoring network. 

The findings are of significant importance to conservation, management, and people-elephant conflict, particularly where major herd movements and migrations are predicted. 
